# High-Accuracy 3-D Sensor for Rivet Inspection Using Fringe Projection Profilometry with Texture Constraint

**DOI:** 10.3390/s20247270

**Published:** 2020-12-18

**Authors:** Yunfan Wang, Huijie Zhao, Xudong Li, Hongzhi Jiang

**Affiliations:** Key Laboratory of Precision Opto-Mechatronics Technology, Ministry of Education, School of Instrumentation and Optoelectronic Engineering, Beihang University, Beijing 100191, China; wangyunfan@buaa.edu.cn (Y.W.); hjzhao@buaa.edu.cn (H.Z.); xdli@buaa.edu.cn (X.L.)

**Keywords:** fringe projection profilometry, high dynamic range, rivet inspection, diameter measurement, 3-D shape measurement

## Abstract

Riveted workpieces are widely used in manufacturing; however, current inspection sensors are mainly limited in nondestructive testing and obtaining the high-accuracy dimension automatically is difficult. We developed a 3-D sensor for rivet inspection using fringe projection profilometry (FPP) with texture constraint. We used multi-intensity high dynamic range (HDR) FPP method to address the varying reflectance of the metal surface then utilized an additional constraint calculated from the fused HDR texture to compensate for the artifacts caused by phase mixture around the stepwise edge. By combining the 2-D contours and 3-D FPP data, rivets can be easily segmented, and the edge points can be further refined for diameter measurement. We tested the performance on a sample of riveted aluminum frame and evaluated the accuracy using standard objects. Experiments show that denser 3-D data of a riveted metal workpiece can be acquired with high accuracy. Compared with the traditional FPP method, the diameter measurement accuracy can be improved by 50%.

## 1. Introduction

For the manufacturing of large thin-wall structures, hundreds of thousands of rivets are installed in lap joints, which are subject precise inspection. The fatigue and crack around or inside the rivet generated after riveting jeopardize craft safety and integrity [1,2]. Many riveting parameters must be examined for quality control besides crack detection, and rivet dimension is one of the key parameters that indicates the fatigue performance of lap joints [3,4]. Specifically, the geometric feature of the rivet head assists the estimation of the applied squeezing force, whereas the height of rivet indicates the interference value [5,6]. The accurate dimensions of rivets and riveted workpieces will feedback to the manufacturer for process optimization and mechanical study. Therefore, a high-accuracy rivet inspection sensor is highly needed.

Nondestructive measurements are mainly applied to rivet inspection. However, current techniques are mainly limited in crack detection and unable to obtain rivet dimensions. Based on the electromagnetic induction principle, eddy current (EC) inspection detects subsurface damages using probes, such as sensing coils [7], hall sensor [8], giant magneto-variance (GMR) sensor [9], or magneto-optic imaging sensor [10]. Although the EC-based technique can automatically measure far-side cracks, sensing the crack in rivets is difficult and inspection is time consuming. Ultrasonic inspection is another option for rivet crack sensing. The crack is detected using ultrasonic signal frequency analysis and the depth of the crack can be estimated using time of flight (ToF) [11]. This kind of system needs additional sensors to locate the rivet and is therefore difficult to be automated. Machine vision technology has also been used to locate and inspect rivets from 2-D images [12,13]. Liu et al. developed an enhanced visual inspection system named Edge of Light^TM^ (EOL) to successfully locate rivets and inspect hidden corrosion damages [13]. By using a specially designed rectangular band light source, the surface slope can be converted into light intensity and the algorithm is performed on the captured image to detect rivets under complex light conditions induced by illumination and workpiece surface reflectivity variance. However, the aforementioned techniques lose the topographic information of the rivet because only 2-D images can be captured. Xie et al. used a commercial 3-D laser scanner to obtain the 3-D data of riveted aircraft skin and then performed multiple structure fitting to detect the rivets. Nevertheless, the quality of the measured point cloud is poor, which suffers from severe noises, missing contours, and density anisotropy [14].

Fringe projection profilometry (FPP) has been widely used in 3-D shape measurement due to its merits of measuring dense 3-D data with high accuracy and speed [15,16]. It enables numerous industrial workpiece measurements, such as composite panel [17], honeycomb core structures [18], and thermal forging parts [19], and other applications [20,21,22]. If dense 3-D data is obtained, dimension parameters and surface cracks can be easily extracted. However, for non-diffuse and stepwise objects, the measurement accuracy is seriously affected [16]. The glitter of the metal surface can generate a saturated region [23], whereas the stepwise shape results in phase mixture along sharp edges [24]. Unfortunately, a riveted workpiece has a shiny surface with non-uniform reflectance and the rivet itself has a cylindrically stepwise shape. Therefore, traditional FPP cannot be directly transplanted for precise rivet inspection application. According to our previous research [23], high dynamic range (HDR) FPP can address the problem of various reflection characteristics; however, the current methods only focus on HDR fringe pattern acquisition and the HDR texture cannot be obtained. Furthermore, through our observation, the texture image contains clear edge information. If HDR texture can be utilized, it has the potential to improve edge reconstruction and complete this measurement task.

To overcome these barriers, an improved FPP sensor is developed combined with texture constraint to measure high-accuracy rivet 3-D data and inspect basic dimension parameters, such as diameter and height. A revised multi-intensity method is adopted to achieve HDR 3-D shape measurement and HDR texture merging. Additional constraint from the merged texture image helps refine the data around the stepwise rivet edge and compensate for the 3-D edge data. The texture information is also used to segment 3-D points and calculate rivet diameter and height. The experiment verified that our sensor can achieve high-accuracy measurement.

The remainder of this paper is organized as follows: Section 2 explains the principle of our sensor, including HDR FPP measurement and rivet edge refinement. Section 3 shows the experimental results of dimension measurements proving the accuracy and practicality. Section 4 discusses the results, while Section 5 summarizes this paper.

## 2. Methods

A riveted workpiece requires a highly automatic 3-D shape measurement, and FPP can complete this task with high accuracy. However, the strong reflection generated from the metal surface and the discontinuity caused by the stepwise edge will degrade the measurement quality. An improved FPP method with texture constraint is presented to achieve HDR and highly accurate measurement.

**Figure 1:**
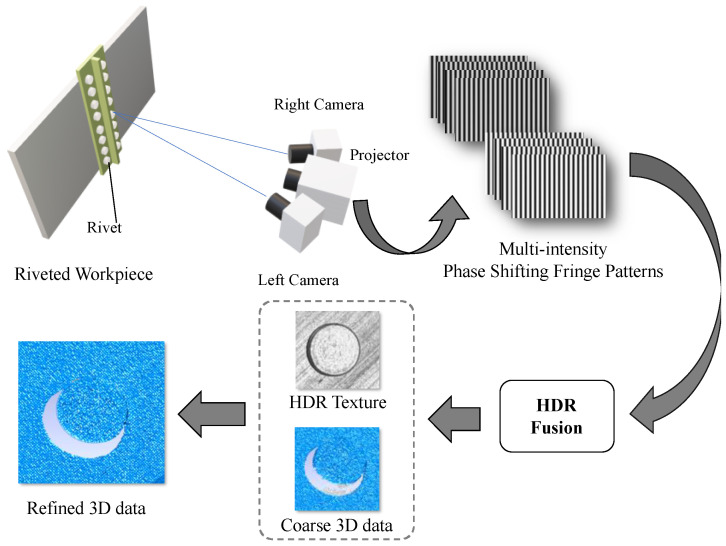
Scheme of the proposed method where an improved high dynamic range fringe projection profilometry (HDR FPP) method with texture constraint is used. The basic idea is to combine the 3-D FPP data with 2-D texture to achieve better performance on rivet inspection.

Figure 1 illustrates the scheme of our proposed method. A dual camera FPP sensor is used, where four-step phase-shifting fringe patterns are projected onto the workpiece surface while both cameras record the fringe images simultaneously. To address the strong reflection, sets of fringe patterns with different projected intensities and exposure times are acquired. These multi-intensity fringe patterns are then fused to obtain HDR fringe patterns and HDR texture separately. HDR fringe patterns are used to reconstruct coarse 3-D points, while the HDR texture is used to provide additional constraint for stepwise edge refinement and artifact elimination. By combining the texture information, the refined 3-D data can be obtained, and dimension parameters can be automatically measured with high accuracy.

In this section, the basic principle of four-step phase-shifting FPP is briefly reviewed. Then, the HDR method for 3-D shape measurement and texture merging are introduced. Finally, the texture constraint for rivet edge refinement and outlier removal are discussed.

### 2.1. Four-Step Phase-Shifting FPP

Fringe projection profilometry is adopted as the main 3-D data acquisition method and a four-step phase-shifting algorithm is applied owing to its leverage of accuracy and speed. The basic principle of the phase-shifting technic is to project a sequence of phase-shifting sinusoidal fringe patterns onto the object surface so that the phase information modulated by the object shape can be retrieved from the captured images point by point. For the four-step phase-shifting algorithm, the projected fringes can be formulated as:

(1) $$I_{1}(x,y)=a(x,y)+b(x,y)\cos(\phi (x,y)),$$

(2) $$I_{2}(x,y)=a(x,y)+b(x,y)\cos(\phi (x,y)+\frac{\pi }{2}),$$

(3) $$I_{3}(x,y)=a(x,y)+b(x,y)\cos(\phi (x,y)+\pi ),$$

(4) $$I_{4}(x,y)=a(x,y)+b(x,y)\cos(\phi (x,y)+\frac{3\pi }{2}),$$

where *a* (*x*, *y*) is the average intensity, *b* (*x*, *y*) represents the intensity modulation, and *ϕ*(*x*, *y*) is the wrapped phase, which can be solved from the following equation:

(5) $$\phi (x,y)=\arctan\left[\frac{I_{4}(x,y)-I_{2}(x,y)}{I_{1}(x,y)-I_{3}(x,y)}\right],$$

where *I_i_* (*x*, *y*) is the recorded image.

The solved phase is wrapped in the range of [−π, π] due to the use of the arctangent function. The multi-frequency method or other temporal absolute phase retrieval method can be further applied to determine the fringe order and remove 2π discontinuities [25]. The unwrapped continuous phase map is then used for stereo matching and triangulation-based 3-D reconstruction.

For the dual camera setup, each camera can be molded as:

(6) $$s\left[\begin{array}{c} u\\ v\\ 1 \end{array}\right]=A\left[R|t\right]\left[\begin{array}{c} X\\ Y\\ Z\\ 1 \end{array}\right],$$

or for simplicity:

(7) sp=MX,

where (*X*, *Y*, *Z*) are the coordinates of the 3-D point in world coordinate space, (*u, v*) are the coordinates in the image space, *s* is the scale factor, **A** represents the intrinsic matrix of the camera, and [**R|t**] represents the extrinsic matrix. **M** is the 3 × 4 projection matrix, which can be determined through camera calibration [26]. Combining the equation of the left and right cameras, homogeneous coordinates **X** = [*X*, *Y*, *Z*, *1*]^T^ can be solved after substituting **p** with matched image point coordinates. Hence:

(8) $$\{\begin{array}{c} s^{l}p^{l}=M^{l}X\\ s^{r}p^{r}=M^{r}X \end{array},$$

where subscripts *l* and *r* denote the left and right cameras, respectively. Moreover, the world coordinate is aligned with the left camera coordinate for simplification. Figure 1 displays the system setup.

### 2.2. Multi-Intensity HDR FPP Measurement

The reflectance of the rivet head surface largely varies because of its surface roughness and material property. This effect results in an inhomogeneous signal-noise ratio (SNR) of the FPP sensor and causes regional sensor saturation. For most of the industrial riveted workpieces, the problem also occurs on other metal surfaces besides rivet heads. Therefore, HDR measurement is highly necessary. According to our previous research [23,27], HDR 3-D shape measurement can be achieved by merging a sequence of fringe images with different camera exposure times and projected intensities. The exposure time and projected intensity are increased for surfaces with low reflectance, whereas they are decreased for surfaces with high reflectance to avoid saturation.

Assuming *N* sets of exposure time and projected intensity parameters are chosen, the *n*th (*n* = 1, 2, …, *N*) modulation intensity *B* calculated from captured fringe images can be expressed as:

(9) $$B_{n}(x,y)=\{\begin{array}{cc} \frac{\sqrt{{\left[I_{1n}(x,y)-I_{3n}(x,y)\right]}^{2}+{\left[I_{2n}(x,y)-I_{4n}(x,y)\right]}^{2}}}{2} & ,I_{in}<255\;i=1,2,3,4\\ 0 & ,\;\mathrm{otherwise} \end{array},$$

where *I_in_* (*x*, *y*) is the *i*-step fringe image with the *n*th parameters. To select the optimal SNR pixel with the highest modulation, a corresponding mask image is generated by:

(10) $$Mask_{n}(x,y)=\{\begin{array}{l} 1,\;if\;B_{n}(x,y)=\max\{B_{k}(x,y),k=1,\ldots ,N\}\\ 0,\;otherwise \end{array},$$

The image fusion process can be then formulated as:

(11) $$F_{i}(x,y)=\sum_{n=1}^{N}Mask_{n}(x,y)\cdot I_{in}(x,y),\;i=1,2,3,4,$$

where *F_i_* (*x*, *y*) is the *i*-step fused fringe image. The fused fringe images are then utilized for phase retrieval.

The aforementioned technique only achieves HDR phase retrieval; however, the phase mixture caused by the stepwise object still affects the phase-matching accuracy. Therefore, additional constraint must be introduced to compensate for this effect. Through our observation, the texture images contain more edge information that can be used as additional constraints. A texture HDR fusion algorithm is added to obtain a high-quality texture image for rivet edge refinement.

Many 2-D HDR fusion algorithms have been proposed in the computational photography community [28]. In our implementation, the classical Debevec’s algorithm [29] is adopted because it only requires differently exposed images to estimate a response function for image fusion. This algorithm can be easily assembled because, during HDR FPP measurement, the object is stationary when multiple images with different intensity conditions are taken. The difference is that in HDR FPP, not only the exposure time but also the projected intensity is changed.

The first step of HDR photography is to recover the camera response curve *f*, which is based on the reciprocity condition, hence:

(12) $$I_{mn}=f(E_{m}t_{n}),$$

where *I_mn_* is the pixel values, *E* is the irradiance, *t* is the exposure time, and m is the pixel index, while *n* indices are different exposure settings. In HDR FPP, given that the irradiance is proportional to the intensity of the projector light, the irradiance can be denoted as:

(13) $$E_{m}=k_{n}^{P}E_{m}^{R},$$

where $k_{n}^{P}$ is the *n*th projector intensity coefficient and $E_{m}^{R}$ is the relative irradiance. Accordingly, the reciprocity condition can be rewritten as:

(14) $$I_{mn}=f^{R}(E_{m}^{R}K_{n}),$$

where *K_n_* = $k_{n}^{P}$ *t_n_* is the HDR coefficient that is calculated from the projector setting and exposure time. Given that the reciprocity condition remains satisfied, the relative camera response *f^R^* can be then utilized for image fusion [29]. For the FPP system, the texture image for the HDR fusion can be calculated from the fringe pattern, which is expressed as:

(15) $$I_{0n}(x,y)=\frac{I_{1n}(x,y)+I_{2n}(x,y)+I_{3n}(x,y)+I_{4n}(x,y)}{4},\;n=1,2,\ldots ,N,$$

If time is permitted, the texture image can be also obtained by projecting an additional white pattern.

**Figure 2:**
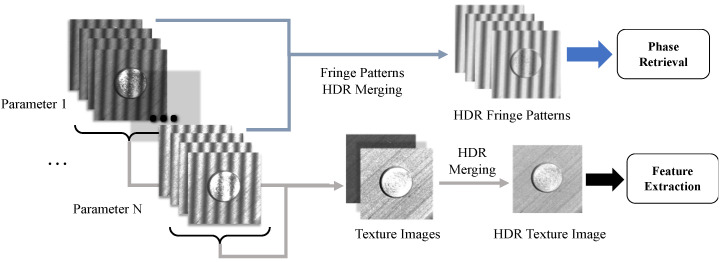
Principle of HDR measurement. Sets of images are recorded under different exposure times and projected intensities. Texture images and fringe pattern images are separately merged to achieve HDR measurement. The HDR patterns are then used for phase retrieval while HDR textures for feature extraction.

Figure 2 illustrates the principle of the HDR measurement. The HDR fringe patterns are then used for phase retrieval and FPP 3-D reconstruction, which is introduced in Section 2.1, while the HDR texture is further processed to obtain circle features for edge refinement. In practice, the HDR procedure is summarized as:

1. Determine a set of HDR parameters, including the exposure time and projected intensity.
2. Set the *i*th parameter.
3. Project and capture a sequence of fringe patterns.
4. Set the *i*+1th parameter. Repeat Steps 3–4 until no more parameters remain.
5. Merge HDR fringes by using Equations (8)–(10) and calculate the 3-D points.
6. Calculate the texture images from fringe patterns by using Equation (15). Merge HDR texture images on the basis of HDR coefficients and compensate for the 3-D points with texture constraint.

### 2.3. Rivet Edge Refinement based on Texture Constraint

The texture information in the FPP 3-D sensor has been long neglected. Nonetheless, through our observation, texture images preserve more edge information than the phase map, which can compensate for the 3-D data where surfaces are discontinuous. Specifically, for the FPP method, the phase mixture will degrade edge reconstruction. However, the gradient change around the edge in the texture map is well-preserved, which can improve the accuracy. These 2-D features detected in the texture image of FPP are the texture constraints.

In our method, the HDR texture images provide constraints for refining the sharp edge of the upper rivet surface and eliminate outliers between the rivet head and bottom surfaces. We assume the edge of the squashed rivet can be fitted as an ellipse, so this algorithm only focuses on the ellipse feature.

This procedure comprises three steps:

1. Rivet contour extraction—the rivet contours are extracted as constraint from HDR texture images and approximated as ellipses.
2. Edge reconstruction—rivet contours from the left and right cameras are correspondingly paired, which are then reconstructed as 3-D points.
3. Outlier elimination—the outliers generated by phase mixture are eliminated.

The detailed operations are described in the following subsections.

#### 2.3.1. Rivet Contour Extraction

The aim of this step is to obtain the rivet segmentation and ellipse fitted rivet contour. First, we used the Hough transformation algorithm [30] to locate rivets in the HDR texture and segmented 1.5 scale region as the region of interest (ROI) for the further process. Then, for each ROI, we used the Canny detector [31] and interpolation method to extract the edge map in subpixels. Finally, we performed the ellipse extraction algorithm to obtain the fitted contour and generate a set of sample points of the fitted ellipse for reconstruction.

For ellipse extraction, many matured algorithms can be used [32,33]. In our practice, we first eliminated the short contours produced by the Canny detector. Then, to sift through remains, we fitted each contour as an ellipse, and a group of thresholds, including the ratio of the long and short axes, area, and average intensity, are set to examine the candidate contours. Thereafter, the only true edge of rivet can be selected. Finally, ellipse fitting is applied to the rivet edge and a set of subpixel sample points are generated.

#### 2.3.2. Edge Reconstruction and Refinement

The simplest way to reconstruct a rivet edge is to directly use a binocular stereo vision algorithm given that left and right image contours have been extracted. However, occlusion and slight misalignment induced by a perspective difference may affect the matching process. In such a case, the left camera actually preserves a clearer and more accurate left edge of the rivet, whereas the right camera preserves the right. Therefore, reconstructing separately and then combining the results is acceptable. One possible solution for addressing occlusion is to regard the binocular FPP system as two separate monocular systems and fuse the data; however, this technique requires projector calibration, which is hard to implement when the camera resolution is significantly higher than that of the projector. We propose using FPP 3-D data as priors to overcome projector calibration.

**Figure 3:**
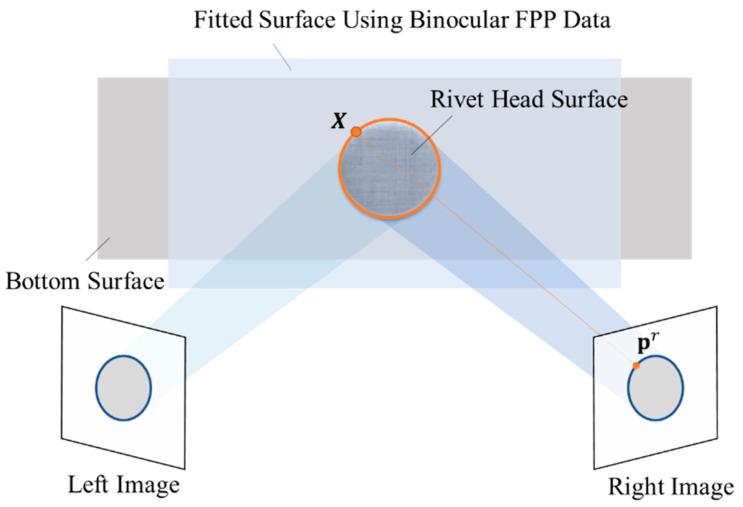
Principle of edge reconstruction by combining dual camera FPP 3-D data and monocular image contour. The orange line illustrates the 3-D coordinates solving of the right camera, where the spatial point **X** is the intersection between fitted plane and camera ray. The same procedure can also be applied to the left image.

We assume the squashed rivet surface is a plane after mechanical processing. Given that FPP has reconstructed coarse 3-D points, the upper rivet head surface can be segmented according to texture coordinates. First, the rivet contours from dual cameras and 3-D segmentation are paired on the basis of phase map and epipolar constraint [34]. We then fitted the upper surface of the rivet head as a plane and points of spatial ellipse can be solved from each camera image by:

(16) $$\{\begin{array}{c} n\cdot {\left[x-x_{0},y-y_{0},z-z_{0}\right]}^{T}=0\\ sp^{i}=M^{i}X \end{array},$$

where **n** is the plane norm, (*x*_0_, *y*_0_, *z*_0_) is a plane point, and subscript *i* can be *r* or *l*, which represents right or left cameras, respectively. Finally, after we reconstructed the sets of the edge points from both cameras, we refitted the spatial ellipse in the plane using the union set and obtained the refined edge. Figure 3 illustrates the principle of this method.

#### 2.3.3. Outlier Elimination

In the FPP-based 3-D shape measurement, the stepwise surface can cause phase mixtures and generate outliers between the upper and bottom surfaces. Given that the texture and fringe images have the same coordinates, the upper and bottom surfaces are segmented using the texture image. The planes are fitted accordingly, and the plane norms are converted into the same direction. A distance threshold is used to determine whether the point is within one of the planes of not, which can be formulated as:

(17) $$Mask(p)=\{\begin{array}{l} 1,\;if\;\left|h_{u}\right|<h_{s}\;or\;\left|h_{b}\right|<h_{s}\\ 0,\;otherwise \end{array},$$

where *h_u_* and *h_b_* is the distance to the upper and bottom plane, and *h_s_* is the distance threshold. According to this mask, all the points with zero are eliminated. After this step, the rivet diameter can be estimated on the basis of the spatial ellipse, while height can be calculated according to the distance between the center point and rear surface.

## 3. Experiment

To verify our principle, we first test the performance of the HDR measurement for a sample of the real riveted workpiece. Then, we conduct an accuracy evaluation for diameter and height measurement on metal standard objects.

**Figure 4:**
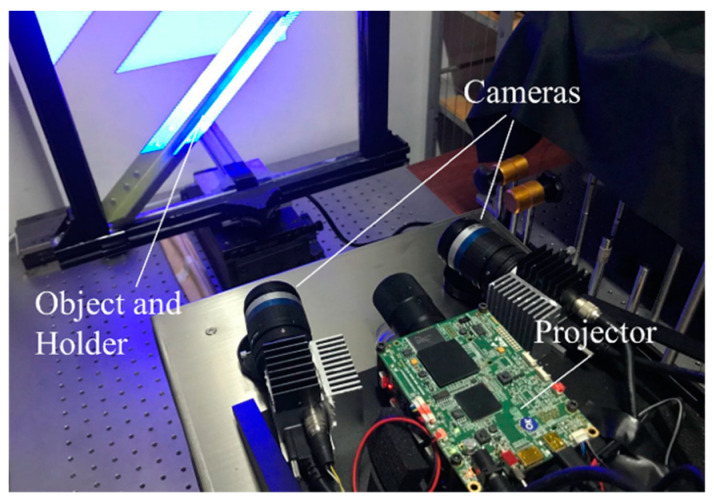
Experiment setup.

The experiment setup depicted in Figure 4 comprises two CMOS cameras (acA5472-17 um/uc, Basler, Ahrensburg, Germany) with a 5472 × 3648 resolution, Moritex ML-U2515SR-18C lenses with a 25-mm focus length, and a blue light DMD-based projector with a 1280 × 800 resolution. The projector is set in the middle of the system and the angle between two cameras is roughly 24 degrees. The working distance of our sensor is 400 mm. A multi-frequency method [25] is used to unwrap the phase and the fringe periods are set at 13, 14, and 15 pixels.

### 3.1. HDR Measurement

As mentioned in Section 2.2, the basic assumption is the reciprocity of projected intensity and exposure time. We first verify the reciprocity assumption on the basis of the performance on HDR texture fusion. Then, a sample of the riveted workpiece is tested for the HDR 3-D shape measurement.

#### 3.1.1. HDR Texture Image Fusion

The HDR parameters used in this experiment are shown in Table 1. Given that the metal roughness of the rivet head generates non-uniform reflectance, it requires more parameter settings to avoid missing points that further degrade crack detection. Furthermore, the changing projector than the camera is preferable because high -intensity projection is normally faster than high exposure time photography.

**Figure 5:**
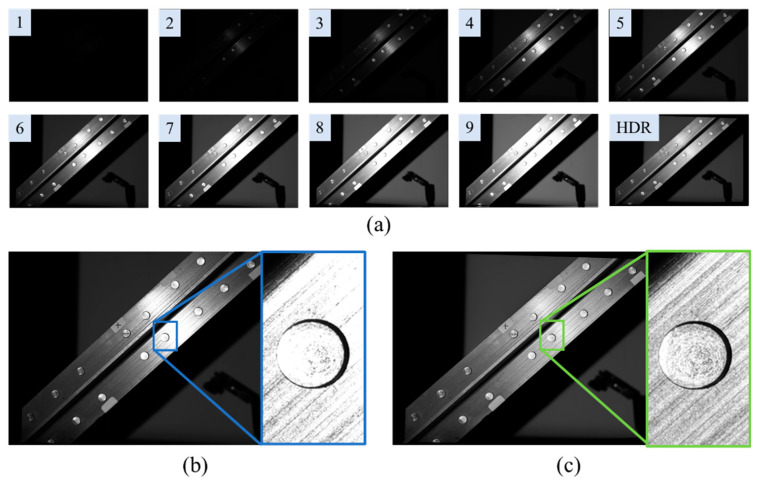
HDR texture fusion. (**a**) demonstrates the texture images of different projected intensities and exposure times. (**b**) is the texture image under 80 projected intensity and 33.333 ms exposure time. (**c**) is the HDR merged texture under the parameter settings in Table 1.

The textures generated from fringe patterns and the fused HDR texture are demonstrated in Figure 5a. The HDR texture verified the reciprocity remains satisfied by the changing exposure time and projected intensity. Figure 5b illustrates a texture image under a single parameter, which keeps most of the regions unsaturated; however, there remains strong metal reflection in the highlights region. Contrastingly, the HDR method in Figure 5c preserves most of the region unsaturated, which verifies the assumption. Figure 5c shows that the edges and textual details are well preserved, which provides additional constraints for edge refinement.

#### 3.1.2. HDR 3-D Measurement for Riveted Workpiece

The 3-D measurement for the sample of the riveted workpiece is also conducted. The object is a segment of metal frame with 18 aluminum rivets. The HDR parameter settings used in this experiment are the same as in Table 1.

**Figure 6:**
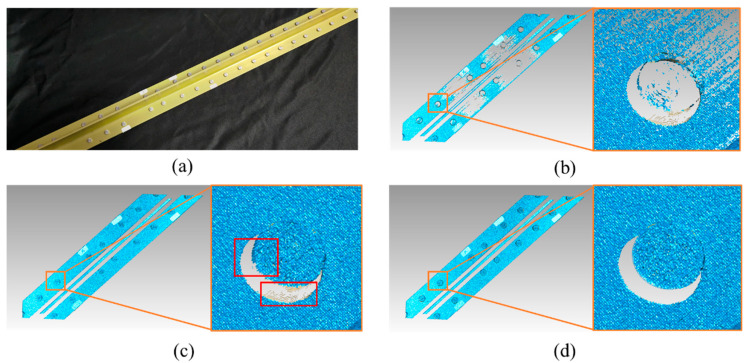
Measurement results of the riveted frame. (**a**) is the sample of the riveted workpiece, which is a riveted aluminum frame. (**b**) is the measurement result under the traditional FPP method with a single intensity setting. (**c**) is the measurement result of HDR FPP without texture constraint, where the red boxes indicate the missing points and artifacts. (**d**) is the measurement result under our proposed method.

Figure 6 illustrates the comparation of the 3-D data among the traditional FPP method, HDR FPP method [23,27], and the proposed principle using texture constraints. The HDR principle enables denser 3-D data acquisition on a metal surface, whereas texture constraint enhances edge accuracy and eliminates artifacts. Figure 6c presents that noticeable missing data and artifacts exist between edge points and surface points, which indicates that the diameter will be diminished if the algorithm uses the FPP surface data only.

Given that the phase map and texture map share the same image coordinates, the texture constraint can also help further data analysis, such as rivet localization, diameter and height extraction, and crack detection. Specifically, after the spatial ellipse is reconstructed using texture constraint, the center point of the rivet and its diameter can be estimated. Given that the contour of the rivet has been extracted in 2-D texture using the method described in Section 2.3, the periphery bottom surface can be also segmented in the 2-D texture by setting a proper scale. Then, the corresponding 3-D points can be found according to the image coordinates, and the height is calculated as the vertical distance between the center point of rivet surface and the fitted surface of the peripheral frame.

**Figure 7:**
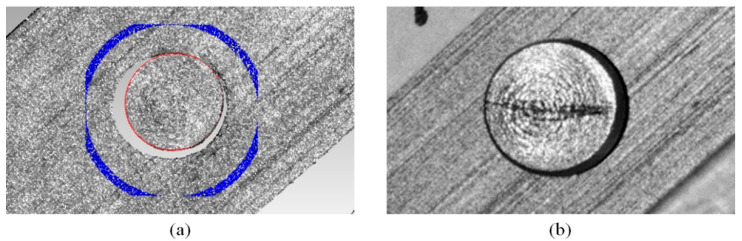
(**a**) Regional 3-D reconstruction of the riveted workpiece by using the proposed method. The red points are the edge compensated by texture constraint, while the blue region is the bottom surface for height extraction. (**b**) the HDR texture of a damaged rivet, where the damage is enhanced by HDR fusion.

Figure 7a demonstrates the regional segmentation of the riveted workpiece, where the red points illustrate the edge points, whereas the blue points are used for bottom surface fitting. In practice, we set the 1.5 to 1.6 scale of the Hough circle as the region of the bottom surface in the texture image. The whole process is automatic, and the edge of the rivet head can be extracted precisely. Figure 7b shows the HDR texture enhances the damage on the rivet surface, which can help further detect cracks. As for the measurement speed, it takes about 1 min to project and record all the images due to the frame rate of the hardware, and 1 min for reconstruction and parameter extraction.

### 3.2. Accuracy Evaluation

Height and diameter were selected as indicators to evaluate our system accuracy of dimension measurement. Given that obtaining the ground truth of the riveted workpiece is difficult, the experiments are conducted on metal gauge blocks to test the height accuracy and standard cylinders are used for diameter accuracy.

#### 3.2.1. Height Accuracy

**Figure 8:**
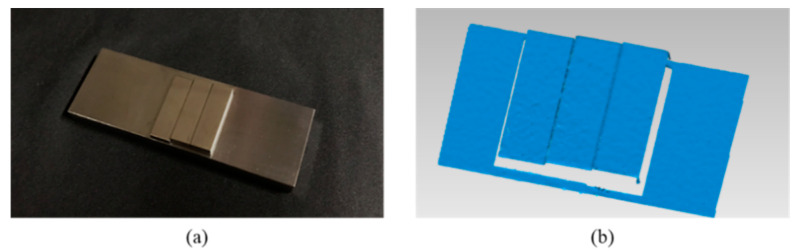
Experimental results on gauge blocks. (**a**) demonstrates the gauge block standards. (**b**) illustrates the measured 3-D data.

The gauge blocks with different thicknesses were assembled as combined standards for height measurement. The height is the distance between the front and rear fitted surfaces, which apparently should be equal to the thickness of the front block. Measurement results are shown in Figure 8 and Table 2. As can be seen in the results, the height measurement error is within 10 um.

#### 3.2.2. Diameter Accuracy

Six standard cylinders are chosen for diameter measurement. The diameters of the cylinders are selected as 4, 5, 6, 7, and 8 mm (tolerance ± 0.001 mm), respectively, to simulate the scale of the rivet. Given that the none-HDR method failed to acquire enough data for diameter measurement, only the method between the traditional HDR method [23,27] and our proposed method is compared. For the traditional FPP method, the diameter is extracted by fitting the most outside points, which uses FPP 3-D points only. In contrast, the proposed method utilizes the texture constraint.

**Figure 9:**
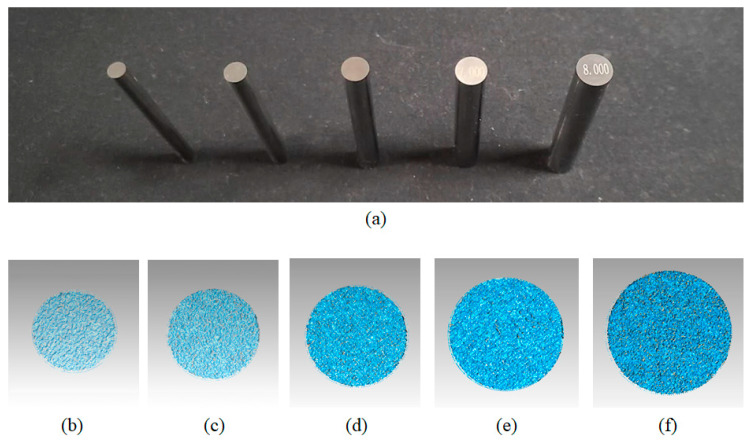
Experimental results on standard objects. (**a**) demonstrates the cylinder standards. (**b**–**f**) illustrate the measured 3-D data of the end surface: (**b**) 4.000 mm, (**c**) 5.000 mm, (**d**), 6.000 mm, (**e**) 7.000 mm, (**f**) 8.000 mm.

The measurement results are shown in Figure 9 and Table 3. The diameter measurement is more accurate than the traditional FFP method without texture information. The traditional FPP method tends to diminish the diameter extraction. Contrarily, our proposed method improved the diameter measurement accuracy by 50%, which means the texture constraint successfully compensates for the FPP method and the circular feature can be easily extracted.

## 4. Discussion

In this section, the texture constraint and then the rivet inspection are first discussed on the basis of the experiments in Section 3. Finally, the limitation of our principle and future research are discussed.

### 4.1. Texture Constraint

**Figure 10:**
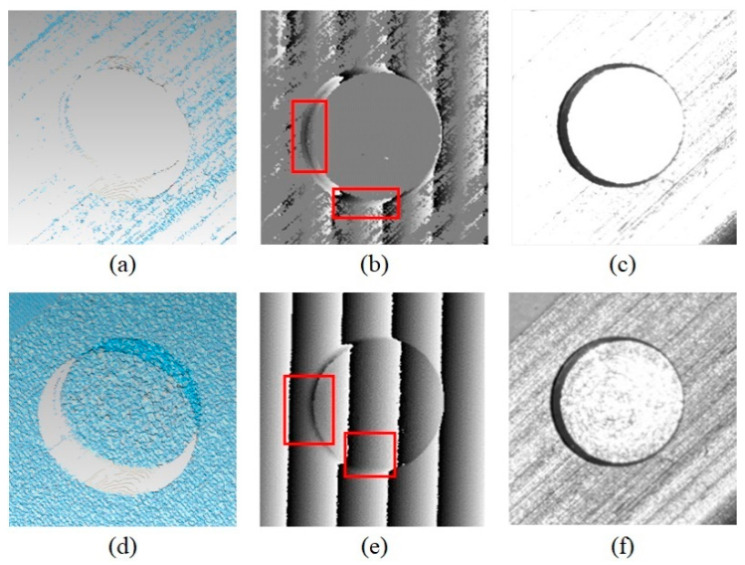
First row is the result of the traditional none-HDR FPP: (**a**) the 3-D reconstruction, (**b**) the wrapped phase of right camera, and (**c**) the texture; The second row is the result of the HDR method without texture constraint: (**d**) the 3-D reconstruction, (**e**) the wrapped phase of right camera, and (**f**) the HDR texture. The red boxes are the phase mixture.

Given that FPP is categorized as an active vision method that can measure surfaces even with no texture, the texture information is usually discarded. However, this information can actually provide additional constraints that overcome the phase mixture caused by stepwise discontinuity. Figure 10 demonstrates the measurement result of the FPP sensor without texture constraint. As can be seen in the wrapped phase, the mixture exists in the none-HDR and HDR FPP methods. Given that the traditional FPP method is dependent on this phase map, the diameter extraction on 3-D data tends to be affected by missing points. Contrastingly, the edge information is well preserved in the gradient of the texture image, which can compensate for the edge reconstruction.

Moreover, many non-plane industrial workpieces normally have textures at discontinuous regions with typical features, such as line, circle, and ellipse. The texture images contain useful clues for 3-D reconstruction and 3-D feature extraction. The aforementioned experiments confirmed the accuracy of measuring an object with a cylinder stepwise shape, which is not only limited in the rivet. It can be inferred that other typical shape features can be categorized as priors and then enhanced by the same principle.

The ability to simplify 3-D feature extraction should be noted. As shown in the principle, rivets are located on the basis of the 2-D texture rather than 3-D points. As far as we know, implementing algorithms that directly run on unstructured 3-D points is difficult, especially on large industrial 3-D point clouds. Contrarily, because 2-D features have already been extracted, segmenting them before reconstruction is more convenient if shape priors are known. This assumption particularly works for workpieces designed in 3-D.

### 4.2. Rivet Inspection

The experiments in Section 3 focus on dimension measurement. The cylinder stepwise shape and strong metal reflectance are overcome. The basic results have been demonstrated, but two points should be discussed further.

In the diameter experiments, we did not directly reconstruct the real contour of the rivet edge but used a fitted spatial ellipse. The reason is that even if real edge points are reconstructed on the basis of the extracted contour in texture, the 3-D fitting processing remains needed for diameter estimation, but the 2-D fitting is normally faster. The procedure depends on the aim of inspection. For measurement, such as the roundness, the true contour must be reconstructed; however, it is less important for diameter.

This principle provides a possibility for robust hybrid crack detection. Figure 7b illustrates that the HDR method enhanced the damage in the image. This detail indicates that, compared with traditional 2-D detection, recognizing the damage in HDR image is easier. Furthermore, the illumination mainly generated by the projector stabilizes the light condition of image formation. If combining 2-D and 3-D data, crack detection can be more robust.

### 4.3. Limitation and Future Research

The principle mainly depends on two steps, namely, the HDR method and texture constraint. One of the limitations is that multi-intensity HDR measurement increases the number of taken images. The speed of rivet measurement is limited by intensity levels and the field of view (FOV). Although FPP can parallelly inspect more rivets within a large FOV, the speed will still be slowed down if numerous intensity levels are needed. Therefore, the high-speed HDR method is necessary.

The rivet should be segmented first and then contours extracted. However, the texture-based method is less robust than the phase-based method. A more elaborate phase compensation algorithm is helpful. Meanwhile, many image segmentation algorithms have been proposed in computer vision communities. Promising deep learning technology, such as Mask-RCNN [35], can also be introduced on this step to address the complex segmentation and contour extraction intelligently. Generally, texture constraint enables the link between 2-D and 3-D data in fringe projection profilometry. More advanced 2-D+3-D algorithms can be investigated in the future.

The experiment results show the successful application on rivet inspection. Our sensor focuses on rivet dimension and surface damage, which can be used as the primary inspection and rivet location but combined with other non-destructive sensors as multimodal instruments, more systematic, automatic, and robust inspection for the riveted workpiece can be achieved.

## 5. Conclusions

We proposed a 3-D sensor for rivet inspection on the basis of improved FPP with texture constraint. By changing the exposure time and projected intensity, we applied an HDR method to overcome strong metal reflectance, which can obtain denser 3-D points and clear HDR texture. Using the extracted features from HDR texture as constraint, edge refinement of the rivet can be achieved. Experiments proved the feasibility and high accuracy. The rivet dimension can be extracted automatically and, compared with the traditional method, the diameter accuracy can be improved by 50%.

The current sensor we proposed mainly inspects dimension, deformation, and superficial damage on the basis of the hybrid data of 2-D texture and 3-D points. Combined with other far-side non-destructive detection sensors, inspecting more parameters and hidden damages of the riveted workpieces is possible. The multimodal sensor can be investigated in the future for rivet inspection. On the viewpoint of 3-D shape measurement, texture information enables the interaction between 2-D and 3-D data, where texture image processing is vital to the compensation. Ultimately, accurate and intelligent algorithms can be applied to improve the robustness of the proposed principle in future works.

## Figures and Tables

**Table 1:** Parameter settings of the HDR measurement.

Serial No.	Projected Intensity ^1^	Exposure Time (ms)	HDR Coefficients
1	43	16.666	0.717
2	45	33.333	1.500
3	50	33.333	1.667
4	60	33.333	2.000
5	70	33.333	2.333
6	80	33.333	2.667
7	100	33.333	3.333
8	125	33.333	4.167
9	150	33.333	5.000

^1^ The intensity setting in projector driver.

**Table 2:** Experimental data on metal gauge blocks.

Materials	Height (mm)	Measured Height (mm)	Error (mm)
Metal	3.000	2.991	0.009
	4.000	3.994	0.006
	5.000	4.997	0.003

**Table 3:** Experimental data on metal cylinders.

Diameter (mm)	Traditional Method		Proposed Method	
	Measured Diameter (mm)	Absolute Error (mm)	Measured Diameter ^1^ (mm)	Absolute Error (mm)
4.000	3.956	0.044	4.022	0.022
5.000	4.902	0.098	4.988	0.012
6.000	5.920	0.080	6.029	0.029
7.000	6.900	0.100	7.013	0.013
8.000	7.887	0.113	7.989	0.011

^1^ Measured diameter is the average of long and short axis.
